# Phototactic and Chemotactic Signal Transduction by Transmembrane Receptors and Transducers in Microorganisms

**DOI:** 10.3390/s100404010

**Published:** 2010-04-20

**Authors:** Daisuke Suzuki, Hiroki Irieda, Michio Homma, Ikuro Kawagishi, Yuki Sudo

**Affiliations:** 1 Division of Biological Science, Graduate School of Science, Nagoya University, Chikusa-ku, Nagoya, 464-8602, Japan; E-Mails: 4dsksuzu@bunshi4.bio.nagoya-u.ac.jp (D.S.); hiro-iri-nuo@mail.goo.ne.jp (H.I.); g44416a@cc.nagoya-u.ac.jp (M.H.); 2 Department of Frontier Bioscience, Hosei University, Koganei, Tokyo, 184-8584, Japan; E-Mail: ikurok@hosei.ac.jp (I.K.); 3 Research Center for Micro-Nano Technology, Hosei University, Koganei, Tokyo, 184-8584, Japan; 4 PRESTO, Japan Science and Technology Agency (JST), 4-1-8 Honcho Kawaguchi, Saitama, 332-0012, Japan

**Keywords:** phototaxis, chemotaxis, transducer, signal transduction, rhodopsin

## Abstract

Microorganisms show attractant and repellent responses to survive in the various environments in which they live. Those phototaxic (to light) and chemotaxic (to chemicals) responses are regulated by membrane-embedded receptors and transducers. This article reviews the following: (1) the signal relay mechanisms by two photoreceptors, Sensory Rhodopsin I (SRI) and Sensory Rhodopsin II (SRII) and their transducers (HtrI and HtrII) responsible for phototaxis in microorganisms; and (2) the signal relay mechanism of a chemoreceptor/transducer protein, Tar, responsible for chemotaxis in *E. coli*. Based on results mainly obtained by our group together with other findings, the possible molecular mechanisms for phototaxis and chemotaxis are discussed.

## 1. Introduction

Microorganisms are subjected to a variety of environmental stimuli to which they respond and adapt. They can show avoidance or attractive behaviors away from or toward stimuli to survive in the various environments in which they live. Light and chemicals are two of the most important signals providing critical information to biological systems, and taxis towards light and chemicals, including some amino acids, are known as phototaxis and chemotaxis, respectively [1,2]. The molecular mechanisms of phototaxis and chemotaxis have been well studied over the past 20 years using various methods [1,3]. Stimulus-induced changes in the motility of cells are well-characterized in bacteria. For chemoreception, motile bacteria sense and respond to extracellular gradients of chemicals by changing their swimming mode to migrate toward more favorable habitats. This behavior, termed *chemotaxis*, has been extensively studied in two bacterial species, *Escherichia coli* and *Salmonella enterica* (for reviews [1,4–14]). Those bacteria have only two swimming modes, run (smooth swimming) and tumble, which result from distinct types of flagellar rotation, counter-clockwise (CCW) or clockwise (CW), respectively. The average durations of run and tumble are about 1 and 0.1 s, respectively. Therefore, the behavior of these bacteria in the absence of a stimulus can be regarded as a three-dimensional random walk. In the presence of a chemical gradient, bacteria modulate their tumbling frequency to seek better habitats.

**Figure 1.**
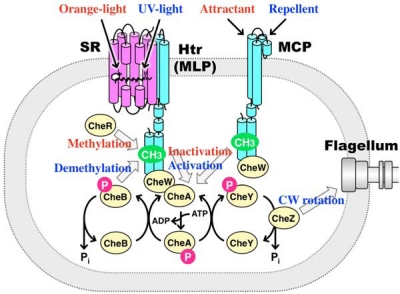
Light and chemical signal transfer cascades in microorganisms. Light stimulation activates sensory rhodopsins (SRs) and triggers *trans-cis* isomerization of the retinal chromophore. Relaxation of the retinal leads to functional processes during the photocycle. SRs transmit light signals to their cognate transducer proteins (Htrs) in the membrane. Htrs form a complex with CheA and CheW, and the complex activates phosphorylation cascades that modulate the autokinase protein, CheY and controls the direction of rotation of the flagellar motor. On the other hand, the cognate chemicals (attractant and repellent) bind to the extracellular domain of the chemoreceptors (MCP) and the binding induces the structural changes of MCP. The signal transfer pathway of MCP is thought to be similar to that of the phototaxis.

The light and chemical stimuli are received by membrane-embedded receptors, Sensory rhodopsin (SR) and Tar, respectively (Figure 1) [15]. Sensory rhodopsin I (SRI) and sensory rhodopsin II (SRII, also known as phoborhodopsin, pR) have 7-transmembrane α-helices with an all-*trans* retinal chromophore (vitamin A-aldehyde). They form 2:2 complexes with their cognate transducer proteins, halobacterial transducer protein for SRI (HtrI) and halobacterial transducer protein for SRII (HtrII) [16,17]. Tar is a two-transmembrane helical protein having a large extracellular ligand-binding domain. Tar, HtrI and HtrII belong to a family of two-transmembrane helical proteins, and Tar and HtrI/HtrII are termed methyl-accepting chemotaxis protein (MCP) and MCP Like Protein (MLP), respectively [1]. The TM2 domains of MCP and MLP are connected to a cytoplasmic helix-turn-helix motif (HAMP domain) [18], followed by a signaling domain which forms a four-helix coiled-coil bundle [19]. MCP and MLP exist as homodimers composed of ∼50–60 kDa subunits [20] and form a signaling complex with a kinase protein, CheA, and an adaptor protein, CheW [21,22]. For the chemo-reception in bacteria, MCP acts not only as a transducer but also as a signal receptor [1], while a direct interaction is required between receptors and cognate transducers for photo-reception in archaea and bacteria [2,3,17]. The signals are transmitted from MCP and MLP to the flagellar motor via a His-Asp phospho-relay [23] from the histidine kinase CheA to the response regulators CheY and CheB. Stimuli-free MCP and MLP activate CheA which phosphorylates its own His residue and then transfers the phosphoryl group to an Asp residue of CheY or an Asp residue of CheB [24–26]. Phosphorylated CheY binds to FliM, a component of the C ring of the flagellar motor, and induces CW rotation of the motor [27,28]. CheZ is a phosphatase which facilitates the dephosphorylation of CheY. The activity of the methylesterase CheB is regulated via its phosphorylation by CheA [29,30], whereas the methyltransferase CheR does not undergo covalent modification. Methylation of MCP reactivates CheA to cancel the attractant response [31].

Light absorption of SRI and SRII triggers *trans*-*cis* photoisomerization of retinal chromophores, leading to cyclic chemical reactions consisting of several sequential intermediate states (K, L, M and O) [32]. The photochemical reaction is referred to as the photocycle. During the photocycle, light signals are transmitted from the SRI-HtrI and SRII-HtrII complexes to the two-component signal transduction cascade consisting of kinases (CheA and CheY), which regulates the rotational direction of the flagellar motor, resulting in positive or negative phototaxis [33,34], while in the case of chemo-reception, the ligand (aspartate) directly binds to extracellular domain of Tar and the binding-signals are transmitted to CheA and CheY, resulting in positive chemotaxis (Figure 1) [1]. It has been reported that HtrII from *Halobacterium salinarum* acts not only as a photo-transducer, but also as a serine chemoreceptor [35]. Thus the light and chemical stimuli are thought to be transmitted to the same phosphorylation cascades in the cytoplasm, and eventually, a phosphorylated response regulator, CheY-P, binds to the C-ring of the flagellar motors which control the direction of the rotation [27]. Flagellar motors are molecular machines powered by the electrochemical potential gradient of specific ions across the membrane, and are classified by the respective coupling ion [36–38]. For some bacteria, such as *Escherichia coli* and *Salmonella enterica*, the flagellar motor is driven by the H^+^ motive force [36], while alkalophilic *Bacillus* and marine *Vibrio* species are driven by the Na^+^ motive force [37]. In this review, based on results obtained mainly from our groups together with other findings, we present and discuss recent progress in the fields of phototaxis and chemotaxis.

## 2. The Signal Transfer Mechanism of the SRII-HtrII Complex

**Figure 2.**
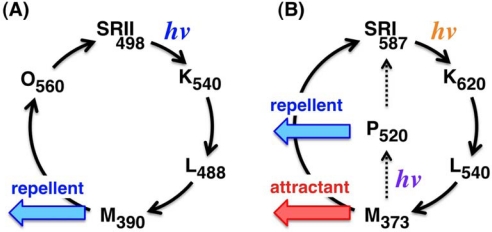
Photochemical reaction cycles of SRII (A) and SRI (B). SRII absorbs blue light and forms K (K_540_), L (L_488_), M (M_390_), and O (O_560_) intermediates [53]. The M and O intermediates are thought to be active states. SRI absorbs orange light and forms K (K_620_), L (L_540_) and the long-lived M intermediates (M_373_), which forms the P intermediate (P_520_) upon the second photon absorption in the near-UV region [54].

In archaeal and bacterial membranes, SRII can be activated by blue light (∼500 nm) and positively regulates the phosphorylation of CheA proteins during the photocycle, which induces the rotation change of the flagellar motor through the phosphorylated CheY (CheY-P), resulting in negative phototaxis from harmful near-UV light (Figure 1) [2,33]. The signal relay mechanisms of SRII have been well characterized using various methods because SRII from an archaeon *Natronomonas pharaonis* (*Np*SRII) can be expressed in *E. coli* as a recombinant protein with quite high stability [39]. The expression system and high stability allowed us to use biophysical and biochemical approaches *in vitro*. In fact, the X-ray crystal structures of *Np*SRII and the complex with truncated *Np*HtrII in the inactive states were solved as well as those of the photo-intermediates, K and M [17,40]. As shown in Figure 2, the essential photocycle of SRII is: SRII (498) → SRII_K_ (540) → SRII_L_ (488) → SRII_M_ (390) → SRII_O_ (560) → SRII [41]. The numbers denote wavelengths (in nm) of the maximum absorption, λmax. An explanation of intermediates, such as SRII_K_, SRII_L_, SRII_M_ and SRII_O_, will be given below. On the other hand, the essential photocycle of SRI is: SRI (587) → SRI_K_ (620) → SRI_L_ (540) → SRI_M_ (373) → SRI [2,33]. Here the P520 intermediate is produced by a second photon absorption by the near-UV light. During the photocycle, the M- and P-intermediates are thought to be essential for the positive and negative phototaxis, respectively (Figure 2) [15,42,43].

We have reported that in the ground (inactive) state of the *Np*SRII-*Np*HtrII complex, the λmax of the complex is essentially the same as that of transducer-free SRII [44], which suggests that large structural changes of SRII do not occur due to the interaction with HtrII. Comparison of X-ray structures between SRII alone and the SRII/HtrII complex reveals no essential changes of SRII due to the association [17,45,46]. Nevertheless we detected structural changes of SRII due to the binding to HtrII [47]. We observed a change in the C-terminal helix detected by solid-state NMR spectroscopy [48], an increase in thermal stability [49] and a pKa change of Asp193 from 6.4 to 5.6 [50]. The complex formation also perturbs the helical structure and hydrogen bond of a side chain in SRII [47]. These results suggest that the structure of SRII is affected by the association with HtrII although these are too small to be detected by X-ray diffraction. From solid-state NMR studies, we reported data representing the structural change of HtrII [51,52], and therefore we conclude that the association affects the conformation of both SRII and HtrII. These conformational changes, however, have only a small effect on the photochemistry [55–57]. It should be noted that truncated HtrII (from position at 1st to 114th or at 1st to 159th) was used in these studies because full-length HtrII was not expressed in *E. coli*.

**Figure 3.**
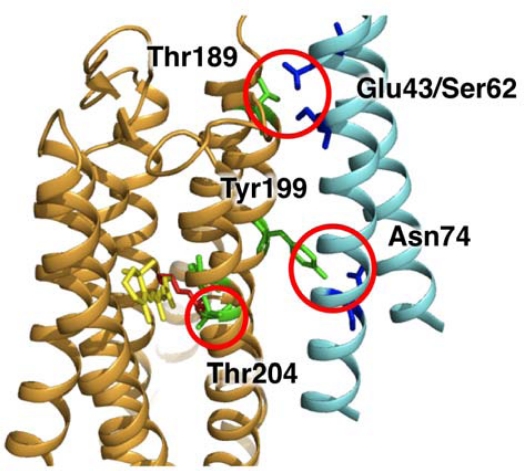
X-ray crystal structure of the SRII/HtrII complex (PDB code 1H2S) [17]. The structure of the ground state of SRII in the complex is very similar to that in the crystal structure of SRII alone. This structure reveals the formation of two specific hydrogen bonds between Tyr199^SRII^ and Asn74^HtrII^ and between Thr189^SRII^ and Glu43^HtrII^/Ser62^HtrII^. The membrane normally is roughly in the vertical plane of this image, and the top and bottom regions correspond to the extracellular and cytoplasmic sides, respectively.

Which helix or residues of SRII and HtrII participate in the binding? Wegener *et al*. reported that the binding surface of SRII consists of helices F and G [58,59]. The crystal structure of the SRII/HtrII complex suggests the formation of two specific hydrogen bonds between Tyr199^SRII^ and Asn74^HtrII^ and between Thr189^SRII^ and Glu43^HtrII^/Ser62^HtrII^ (Figure 3) [17]. To investigate the importance of those hydrogen bonds, Kd values for the binding of various mutants of Bacteriorhodopsin (BR), Halorhodopsin (HR), SRI and SRII with HtrII were estimated by isothermal titration calorimetry (ITC) [60,61]. BR and HR are microbial rhodopsins that work as a light-driven outward proton pump and a light-driven inward Cl^−^ pump without the transducer protein, respectively [62,63]. The Kd value of T189V^SRII^/Y199F^SRII^, the double mutant/HtrII complex, was about 100-fold larger than that of wild-type SRII, whose Kd value was 0.16 mM [60,61]. On the other hand, BR and HR double mutants, P200T^BR^/V210Y^BR^ and P240T^HR^/F250Y^HR^, were able to bind with HtrII, while the wild-type BR and HR did not [61]. Pro200^BR^/Pro240^HR^ and Val210^BR^ /Phe250^HR^ correspond to Thr189^SRII^ and Tyr199^SRII^, respectively. Thus we conclude that those two specific hydrogen bonds play important roles in the binding between SRII and HtrII. In addition, an interaction of the HtrII membrane-proximal domain with the cytoplasmic domain of SRII has been demonstrated by FRET measurements [64,65], by EPR of spin-labels [58,59,66], and by *in vitro* binding of HtrII peptides to SRII [44,67,68]. From these experiments, the stoichiometry of the SRII-HtrII complex is estimated as 1:1, which is in accord with the 2:2 stoichiometry resolved by X-ray structure [17].

**Figure 4.**
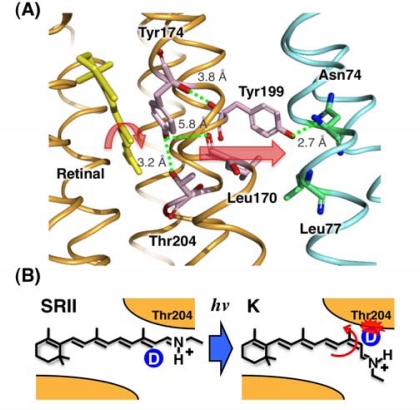
(A) Detail of the SRII-HtrII X-ray structure, which focuses on the midmembrane SRII-HtrII interface containing the core signal relay structure. SRII and HtrII are colored orange and blue, respectively. The numbers in the smaller font are the length between the respective amino acid residues. Using FTIR spectroscopy and photochemical techniques, we and another group reported that Thr199^SRII^ forms a hydrogen bond with Asn74^HtrII^ [56,79]. A functionally important residue, Thr204^SRII^, forms a hydrogen bond with Tyr174^SRII^. Tyr174^SRII^ is also essential for the phototaxis function [81]. (B) The structural change of the retinal chromophore upon formation of the K intermediate of SRII. All seven monodeuterated all-*trans* retinal analogues were synthesized, and the FTIR spectra were measured at 77 K. The enhanced C_14_-D stretch in the K intermediate was assigned as the band originating from the local steric constraint between C_14_-D and Thr204^SRII^ [75,80]. We reported that the band intensity correlated well with the phototaxis signaling efficiency, indicating its functional importance [80].

The properties of early photo-intermediates of the transducer-free SRII photocycle were studied by low-temperature spectroscopy and by flash photolysis at room temperature. Irradiation of SRII below −100 °C produced an intermediate corresponding to the K-intermediate of BR and this intermediate is denoted SRII_K_ [69,70]. We performed comparative studies of transducer-free SRII and BR by means of low-temperature FTIR spectroscopy and obtained invaluable information about SRII alone and the SRII/HtrII complex [47,56,57,70–79]. One of the most interesting and important questions is how SRII transmits the light signal to HtrII, and a clue about that was obtained from the FTIR data. We trapped SRII_K_ at 77 K and compared the SRII_K_ minus SRII spectra in the presence or absence of HtrII [47,78]. This difference spectrum showed clear differences for amide-I and amide-A vibrations, indicating that the complex formation affects the structure of the peptide backbone. In addition, we found that the difference spectra measured in the presence of D_2_O possessed positive and negative vibrational bands of SRII at 3,479(−)/3,369(+) cm^−1^ that were observed only in the presence of HtrII [47]. Of interest, those positive and negative bands disappeared in the T204A mutant while they shifted in the T204S mutant [78]. From these results, we conclude that the bands of 3,479(−)/3,369(+) cm^−1^ originate from the O-H stretch of Thr204 [78]. This indicates that a stronger hydrogen bonding alteration of Thr204 takes place upon the retinal photo-isomerization (Figures 3 and 4). The structural changes are important for the HtrII activation by SRII at the following intermediates. In fact, we reported that mutations of Thr204 and its hydrogen-bonding residue Tyr174 disrupted the negative phototaxis function [81]. Moreover, a single replacement of the corresponding residue can occur in the absence of HtrII. In addition, using deuterated retinal analogs, we demonstrated that a regiospecific steric hindrance between retinal and Thr204 occurs upon formation of the K-intermediate (Figure 4) [75,80]. Although SRII_K_ is considered not to be an active state (signaling state), this BR (A215T) confers weak phototaxis signaling activity [82] and structural changes upon formation of the K intermediate of the A215T mutant are quite similar to the structural changes of SRII [83]. The signaling was greatly enhanced by two additional substitutions, P200T and V210Y, expected to align BR and HtrII in a similar juxtaposition as SRII and HtrII [82], indicating that the BR triple mutant (BR-T) transmits light signals to the motility apparatus through the HtrII protein. In SRII, the three residues form a chain of hydrogen bonds from retinal’s photoisomerized C13 = C14 double bond to residues in the membrane-embedded α-helices of HtrII (Figure 4). These results suggest a chemical mechanism for the signaling that entails an initial storage of the energy of photoisomerization in SRII’s hydrogen bond between Tyr174, which is in contact with retinal, and Thr204, which borders residues on the SRII surface in contact with HtrII, followed by transfer of this chemical energy to drive structural transitions in the transducer helices. The results also demonstrate that evolution accomplished an elegant but simple conversion: The essential differences between transport and signaling proteins in the rhodopsin family are far less than previously imagined. It is noted that Thr204 is an important residue for color tuning and the photocycle kinetics of SRII [84–86], and these observations provide an additional important role of Thr204 in SRII for the negative phototaxis function of the complex [80–83].

Although it has been concluded that the M-intermediate is one of the active (signaling) states from the results of SRII of *H. salinarum* (*Hs*SRII) [87], this is also considered valid for *Np*SRII. Upon formation of the M-intermediate of SRII (SRII_M_), the primary proton transfer occurs from the Schiff base to the proton acceptor [72], Asp75, that is a counterion of the protonated Schiff base (PSB) [88]. Using an SnO_2_ electrode as a pH-sensitive electrode [53], we and Schmies *et al.* demonstrated independently that HtrII-free SRII transports a proton on illumination while the photo-induced proton-pumping activity is lost when SRII binds HtrII [89,90]. We have interpreted those results as follows: at SRII_M_, the cytoplasmic channel (CP) of SRII may be closed by the association with HtrII, and then the proton-pumping is lost. This idea was originally proposed by Spudich, who observed a blockage of the proton flux through the CP when SRI bound its transducer, HtrI [91,92]. Azide and hydroxylamine can react with HtrII-free SRII_M_, and the results show that: (1) azide accelerates greatly and selectively the decay of SRII_M_ that is the reprotonation process of the Schiff base [93], and (2) hydroxylamine attacks the Schiff base mainly at SRII_M_ to bleach the pigment [94]. The reactivity of these water-soluble reagents is a good indicator for the environmental change around the Schiff base. We showed a decrease in the reactivity of these two reagents against the SRII/HtrII complex in comparison with SRII alone [44], which may support the concept of the cytoplasmic closure described above. It is known that at the M- or N-state, photo-dependent structural changes of BR occur at the cytoplasmic ends of helices F and G [95–100]. These movements open a narrow water-accessible channel in the protein, enabling the transfer of a proton from the proton-donating Asp residue to the Schiff base. Similar helix movements of SRII, an outward tilting of helix F, during the photocycle are also suggested by EPR spectroscopy [58] and chemical modification [101]. The photo-induced outward tilting may be hampered by HtrII, which may be critical to the signal transduction. In our study, we measured the temperature dependence of the SRII_M_ minus SRII FTIR spectra in the absence or presence of truncated HtrII at 250–293 K [57]. Significant temperature dependence was observed for the amide-I vibrations of helices only for the SRII/HtrII complex, where the amplitude of the amide-I vibrations was reduced at room temperature. ^13^C-Labeling of SRII or HtrII revealed that such spectral changes of helices originate from SRII and not from HtrII. On the other hand, temperature-dependent structural changes of helices were diminished for the complex of SRII with the G83C and G83F mutants of HtrII. Gly83 is believed to connect the transmembrane helix and the cytosolic linker region in a flexible kink near the membrane surface of HtrII, and its replacement by Cys or Phe abolishes the photosensory function [101]. That study provides direct experimental evidence that Gly83 plays an important structural role in the activation process of the SRII/HtrII complex.

The M-decay rate decreases about 50% when SRII associates with HtrII [55,57]. Hence, the M-decay rates are composed of two components for the mixture of SRII and HtrII that contained free SRII and SRII/HtrII. From analysis of data obtained from mixtures of varying ratios of SRII and HtrII, we were able to estimate a Kd of 15 μM and a stoichiometry of 1:1 [55]. The Kd value of 15 μM is almost 100-fold larger than the dissociation constant in the dark, 0.16 μM [60,67,102], indicating that the association between SRII and HtrII is 100-fold weakened upon the formation of SRII_M_ compared with the ground state. Recently, the weak binding (Kd = 5 μM) was also confirmed using the highly stable M-like state [103]. This change in Kd value may come from the outward tilting of helix F during photocycling. We speculate that the weak binding is caused by the dissociation of the HAMP domain of HtrII from SRII [67].

The nature of SRII_O_ has not been well understood compared with SRII_K_ and SRII_M_, although some characterization experiments have been done. Recently we found characteristic bands at 1,673 (+)/1,656 (−) cm^−1^ in the O-decay accelerated mutants using FTIR spectroscopy [77]. The absorbance of the bands correlated well with the O-decay half-time, which suggests that the helical distortion is important for the acceleration in O-decay. In addition, we demonstrated that characteristic changes in the S-H stretching vibration of the introduced Cys residue was observed in SETC (a SRII quadruple mutant, P182S/P183E/V194T/T204C, showing fast O-decay [84]), which suggests that the structural perturbation near the Schiff base was caused by an acceleration in the O-decay on the extracellular surface. Thus, FTIR analysis of the O-accelerated mutants provided information about the mechanism of the slow O-decay in SRII. The effects on phototaxis and proton pumping are important questions that should be examined because SRII_O_ is one of the active intermediates for signal transduction [87] and is one of the important intermediates for ion-pumping [53].

Using EPR, Wegener *et al.* reported interesting results showing that, in the M-state of SRII, helix F moves outwardly, which causes the rotation of the second transmembrane helix (TM2) of HtrII [58,59]. Those conformational changes may be important for the signal transduction as proposed by Spudich [91]. However, some uncertainties or contradictory points remain. It was reported that the outward tilting of the helices is not affected by the presence of HtrII [59]. It is quite reasonable that the tilting may be hampered by the transducer as was shown by the “cytoplasmic closure” and the reactivities of azide and hydroxylamine. They also described that the tilting continues until the SRII_O_ decay. However, the helix tilting of SRII and the rotation of HtrII were not observed in the crystal structures of the active M-intermediate of the SRII-HtrII complexes [17,40].

**Figure 5.**
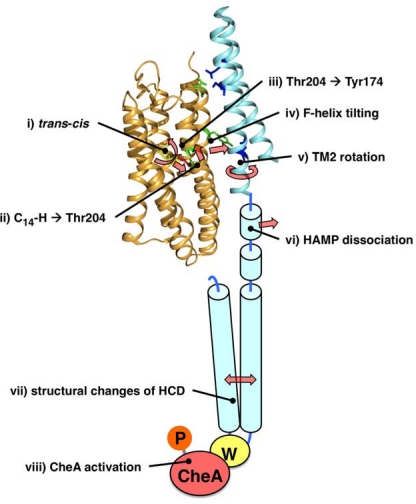
Model for signal transduction mechanism of SRII-HtrII complex.

On the basis of these results, we propose a model for the signal transduction by the SRII-HtrII system (Figure 5) as follows; (i) *trans*-*cis* photoisomerization of the retinal chromophore, (ii) steric hindrance between C14-H of retinal and Thr204, (iii) hydrogen bonding alteration between Thr204 and Tyr174, (iv) F-helix outward tilting of SRII, (v) TM-2 rotation of HtrII, (vi) dissociation of the HAMP domain of HtrII from SRII, vii) structural changes of the Highly Conserved domain (HCD), and (viii) activation (phosphorylation) of CheA. Further structural and spectroscopic analysis of the ground state and the photoreaction intermediates will provide a better understanding of the mechanism for signal transduction by SRII-HtrII. Especially, interactions of CheA and CheW with the SRII-HtrII complex and the signal relay mechanism of the SRII-HtrII-CheW-CheA supra complex will be our next focus.

## 3. Towards Understanding the Signal Transfer Mechanism of the SRI-HtrI Complex

The first clue for identifying the receptor responsible for phototaxis was obtained using BR-lacking mutants of *H. salinarum*. In 1977, the first description of HR was reported by Matsuno-Yagi and Mukohata [104]. However, using a BR^−^ mutant that at that time (in 1983) was believed to express only HR, Tsuda *et al.* found two independent fast and slow photocycles [105]. Bogomolni and Spudich proposed that the pigment having the slow photocycle is the receptor for phototaxis of this bacterium, and proved their hypothesis by isolating a mutant lacking both BR and HR which showed almost the same level of phototaxis as the wild-type [106]. They named this retinal pigment protein sensory rhodopsin I (SRI). Of interest, the ground state of SRI functions as a sensor of positive phototaxis whose action maximum is located at ca. 580 nm while the long-living photo-intermediate absorbing maximally at 373 nm forms the P520 intermediate upon a second photon absorption by the near-UV light [15]. Thus the original state of SRI and its long-lived photointermediate (the M-intermediate) are important for positive and negative phototaxis, respectively. Light > 520 nm can activate the ion pumping rhodopsins, BR and HR, to generate light-energy and cells avoid light of shorter wavelengths which contain harmful near-UV [2,33]. SRI from *H. salinarum* (*Hs*SRI) forms a 2:2 signaling complex with its cognate halobacterial transducer protein (Htr), *Hs*HtrI, in membranes as well as the SRII-HtrII complex [17], and the complex transmits light signals through changes in protein-protein interactions. The excitation light absorbed by *Hs*SRI triggers a *trans-cis* isomerization of the retinal chromophore that is covalently bound to a conserved lysine residue (Lys205) via a PSB linkage [2]. This photoexcitation results in the sequential appearance of various photointermediates followed by a return to the unphotolyzed form of the protein. At its distal end, the SRI-HtrI complex negatively/positively regulates the phosphorylation of CheA during the photocycle, which induces the rotation change of the flagellar motor through the phosphorylated CheY (CheY-P), resulting in positive/negative phototaxis from harmful near-UV light (Figure 1) [2,33]. Thus, SRI has received considerable attention because of its function in mediating opposing signals (On/Off switching of CheA) depending on different colors of light by the photochromic reaction. Compared with SRII, little is known about the molecular mechanism(s) of interactions between SRI and HtrI, about structural changes or about the signal relay mechanism of the phototaxis. One reason is that *Hs*SRI is unstable under various conditions [107], and its inherent instability hampers the elucidation of its molecular mechanism. In fact, although the X-ray crystal structures of BR [108,109], HR [110], SRII [45,46] and the SRII/HtrII complex [17] have been solved over the past 10 years, however almost no structural information at the atomic level has been gained for SRI. In an effort to improve that situation, we cloned and characterized a novel SRI protein from a eubacterium *Salinibacter ruber* (*Sr*SRI), which is the first eubacterial SRI identified as a functional protein [107,111]. *Sr*SRI has all-*trans* retinal as a chromophore, has an absorption maximum at a longer wavelength (557 nm) than does SRII (500 nm), and has a slower photocycle than the light-driven ion pumping rhodopsins (BR, HR and Proteorhodopsin [112]), indicating similarities to the prototypic SRI (*Hs*SRI). *Sr*SRI could be expressed in *E. coli* and shows very high stability, even in detergent micelles, making it possible to prepare large amounts of protein. This also allows preparation of mutant *Sr*SRI, which will allow new approaches to investigate the photo-signaling process in the SRI-HtrI system.

**Figure 6.**
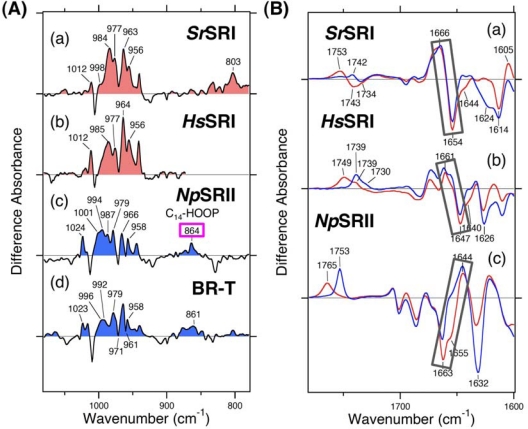
(A) Spectral comparison of the HOOP vibrations of the retinal chromophore upon formation of the K intermediate. *Sr*SRI_K_ minus *Sr*SRI (a) and *Hs*SRI_K_ minus *Hs*SRI (b), difference infrared spectra measured at pH 7.0 and 8.5, respectively. The spectrum of *Hs*SRI was deleted at < 872 cm^−1^. The *Hs*SRI_K_ minus *Hs*SRI spectra are multiplied by 4.2 for the sake of comparison. The samples were hydrated with H_2_O. The SRII_K_ minus SRII (c) and BR-T_K_ minus BR-T (d) difference FTIR spectra are reproduced from reference [70] and [83], respectively, for comparison. (B) Spectral comparison of the amide-I vibration upon formation of the M intermediate. *Sr*SRI_M_ minus *Sr*SRI (a) difference infrared spectra measured at 260 K at pH 7.0 in the 1,780–1,600 cm^−1^ region. The samples were hydrated with H_2_O (red) or D_2_O (blue). The *Hs*SRI_M_ minus *Hs*SRI (b) and SRII_M_ minus SRII (c) difference FTIR spectra are reproduced from reference [54] and [72], respectively, for comparison. Adopted from reference [113].

Utilizing that high stability, we applied FTIR spectroscopy to *Sr*SRI and compared the spectral changes upon formation of the K and M intermediates with those of other archaeal rhodopsins (*Hs*SRI, SRII, BR and HR) (Figure 6) [113]. In the K intermediates, a spectral comparison of the hydrogen out-of-plane (HOOP) vibrations of the retinal chromophore show that the extended chromophore distortion takes place in *Sr*SRI and *Hs*SRI as well as in SRII and BR-T, whereas the distortion is localized in the Schiff base region in BR and HR (left panel in Figure 6) [113]. It appears that the sensor and pump functions are distinguishable from the spectral feature of the HOOP modes. The C_14_-HOOP band at 864 cm^−1^ for *Np*SRII (Figure 6) which is important for negative phototaxis in SRII [75,79] is absent in *Sr*SRI, suggesting that the changes of C_14_-H HOOP are a specific feature among sensors for negative phototaxis. This is reasonable because the counterpart of the C_14_ atom, Thr204 of SRII, is not conserved in other rhodopsins, including SRIs [107]. In the M intermediate, the frequency shifts of amide-I and amide-A vibrations which probe structural changes in the α-helix were opposite between SRI and SRII, where a downshift and an upshift were observed, respectively (right panel in Figure 6). This indicates that the M formation accompanies a weakened hydrogen bond of the α-helix in SRI, but a strengthened hydrogen bond of the α-helix in SRII. In other words, activation of SRI or SRII may involve breakage or formation of the helical structure. The different modes in protein structural changes between SRI and SRII may be correlated with their functional differences.

**Figure 7.**
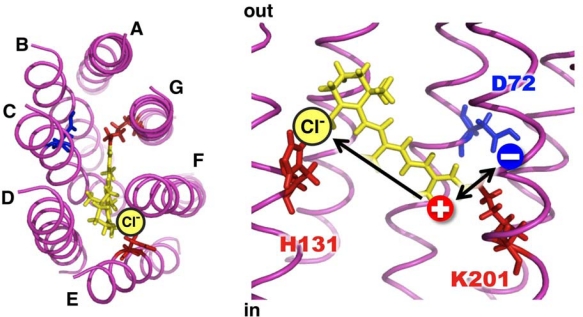
Putative chloride binding site of *Sr*SRI (left, top view; right, side view). The structure was generated using a theoretical model of *Hs*SRI (PDB ID: 1SR1) [121]. It was assumed that a positive charge located on the Schiff base nitrogen is likely to move to the b-ionone ring by chloride ion binding to His131.

Surprisingly, *Sr*SRI is highly stable even in the absence of NaCl, unlike *Hs*SRI [107]. Halobacteria and *Salinibacter ruber* live in highly halophilic environments, suggesting the possibility of effect(s) of salts on the function of SRI. Utilizing the high stability of *Sr*SRI in the absence of NaCl, we examined the effects of Cl^−^ on its photochemical properties. Kitade *et al.* reported the Cl^−^-induced difference attenuated total reflection (ATR) FTIR spectroscopy in the aqueous phase using SRII and they concluded that the binding of Cl^−^ to SRII accompanies protonation of a carboxylic acid [114]. The amino acid was identified as Asp193 because the corresponding band shows a downshift in the D193E mutant protein [114]. However, such effect(s) remain unclear because *Hs*SRI is unstable in dilute salt solutions. We later reported that the absorption maximum of *Sr*SRI is shifted from 542 nm to 556 nm in a Cl^−^-dependent manner with a Kd of ∼300 mM [115]. The bathochromic spectral shift was caused not only by NaCl, but also by other salts (NaI, NaBr and NaNO_3_), implying that the anion (I^−^, Br^−^ and NO_3_^−^) binding site(s) exist in *Sr*SRI [115]. In addition, the photocycling rate was also affected by chloride ion binding. It is well-known that the ion pumping rhodopsins have been optimized to have relatively fast photocycling rates (measured in milliseconds), making them efficient pumps, whereas the sensory receptors, including SRI and SRII, have slow photocycles (measured in seconds), which allows the transient accumulation of long-lived signaling states to catalyze a sustained phosphorylation cascade, including CheA and CheY. To identify the residue(s) involved in the chloride binding, we constructed various mutants of *Sr*SRI and suggested that a conserved residue, His131, is involved in the Cl^−^ binding site [115]. We assumed that a positive charge located on the Schiff base nitrogen moves to the β-ionone ring of the retinal chromophore by chloride ion binding to His131 (Figure 7). The changes of charge distribution result in the following: (i), (ii) and/or (iii) [116–119]: (i) the strength of the electrostatic interaction between the PSB and its counterion or hydrogen bond acceptor; (ii) an alteration in the polarity or polarizability of the chromophore-binding site environment caused by the arrangement of polar or aromatic residues; and (iii) an isomerization around the 6-S bond which connects the polyene chain to the b-ionone ring. Although the high resolution structure of SRI has not yet been solved, it is predicted that the space around His131 is significantly narrow for the Cl^−^ binding, suggesting large structural changes of *Sr*SRI upon Cl^−^ binding. Interestingly, we found that SRI from the archaeon *Haloarcula vallismortis* (*Hv*SRI) exhibited similar alterations due to chloride ion-binding [120]. The binding to *Sr*SRI is likely to be important for the function of the SRI protein family. Thus, as expected, *Sr*SRI can be a key protein to investigate the functions of SRI at the molecular level.

The gene for *Sr*SRI has a downstream sequence immediately followed by a second gene in a probable operon under the control of the same promoter as *Sr*SRI [111]. The putative transducer gene encodes a 518-residue protein with two transmembrane domains at the N-terminal portion followed by an extensive domain with primarily hydrophilic residues. The transducer protein is hereafter called *Sr*HtrI. This topology is similar to that of the Htr transducers from the archaeon *H. salinarum.* The eubacterium, *Salinibacter ruber*, also has two light-driven ion pumps, Xanthorhodopsin [122] and a Halorhodopsin-like protein, making an energy source for living cells. The *Sr*SRI-*Sr*HtrI complex is expected to function as a positive phototaxis sensor against longer wavelengths of light where ion pumping rhodopsins can utilize light energy, and as a negative phototaxis sensor against shorter wavelengths of light which contain harmful UV. Recently we succeeded in producing *Sr*SRI with its cognate full-length transducer protein, *Sr*HtrI, as a fusion construct in *E. coli* as a recombinant protein having all-*trans* retinal as a chromophore for SRI (more than 95% all-*trans* retinal with a small proportion of 13-*cis* retinal), although the expression level was low (0.10 mg/L culture) [123]. The absorption maximum of *Sr*SRI-*Sr*HtrI is at 544 nm, which is shifted from 557 nm in a *Sr*HtrI-dependent manner, implying that *Sr*HtrI interacts with *Sr*SRI and perturbs the retinal chromophore of *Sr*SRI. As described above, the absorption maximum of *Sr*SRI alone is shifted from 542 to 557 nm in a Cl^−^-dependent manner [115], while the Cl^−^-induced spectral red shift (532→544 nm) was also observed in the *Sr*SRI-*Sr*HtrI complex. Thus, absorption maxima of *Sr*SRI (Cl^−^ free), *Sr*SRI (Cl^−^), *Sr*SRI-*Sr*HtrI (Cl^−^ free) and *Sr*SRI-*Sr*HtrI (Cl^−^ free) were observed at 542, 557, 532 and 544 nm, respectively, indicating that the spectral blue shift by *Sr*HtrI-binding (from 557 to 544 nm) is not caused by the effect of Cl^−^.

It was reported that the pKa value of the counterion Asp76 of *Hs*SRI is increased from 7.2 to 8.5 upon association with *Hs*HtrI [124]. To estimate the pKa value of the counterion Asp72 of *Sr*SRI with and without *Sr*HtrI, we performed pH titration experiments. The results indicate that the pKa value of Asp72 is increased from 4.3 to 4.9 upon *Sr*HtrI association [123], although the shift value of the *Sr*SRI-*Sr*HtrI complex (0.6 unit) is smaller than that of *Hs*SRI-*Hs*HtrI (1.3 unit). In the case of SRII, no shift was observed [125]. The lower pKa of *Sr*SRI-*Sr*HtrI (4.9) than *Hs*SRI-*Hs*HtrI (8.5) also indicates that the counterion Asp72 exists in a deprotonated form at neutral pH where the bacteria live, suggesting the functional importance of the deprotonated state of the *Sr*SRI-*Sr*HtrI complex. An important question to understand the signal transfer mechanism is whether the photocycle of *Sr*SRI is affected by the *Sr*HtrI binding. Flash photolysis experiments revealed that the M decay of *Sr*SRI-*Sr*HtrI is 640-fold slower than that of *Sr*SRI alone [123]. This slow photocycle is particularly important because a key difference between transport and sensory rhodopsins is the much slower kinetics of the photochemical reaction cycle of the sensors [2,33]. The effect(s) of binding of CheW, CheA, and CheY on the SrSRI-SrHtrI complex will be the next focus of our study.

Sineshchekov *et al.* reported an interesting study showing that the Schiff base connectivity switch plays an important role in SRI with HtrI [126]. As described above, the PSB plays a essential role in the functions of microbial rhodopsins [2,33], deprotonating in the first half of the photocycle by transferring its proton to an Asp (counterion) residue in the extracellular half-channel and reprotonating in the second half of the cycle from the Asp residue in the cytoplasmic channel. The key step is known as the “Schiff base connectivity switch”, the photoactive site structural change that occurs in the photocycle and changes the accessibility of the retinylidene Schiff base nitrogen from the extracellular to the cytoplasmic channel. In the dark, the SRI-HtrI complex exists in two conformational states, which differ by their connection of the Schiff base in the SRI photoactive site to inner or outer half-channels. In single-quantum photochemical reactions [127], the conformer with the Schiff base connected to the cytoplasmic (CP) half-channel generates an attractant signal, whereas the conformer with the Schiff base connected to the extracellular (EC) half-channel generates a repellent signal. The opposite signals from the two conformers are integrated in the downstream transduction pathways leading to one or the other sign of the behavioral response. In the wild-type complex, the conformer equilibrium is poised strongly in favor of that with CP-accessible Schiff base. The inverting mutations (Glu56 in HtrI; Asn165 and His166 in SRI) [128] shift the equilibrium in favor of the EC-accessible Schiff base form, whereas the suppressor mutations (Asn53 in HtrI; Arg84 and Arg215 in SRI) [128] shift the equilibrium back toward the CP-accessible Schiff base form, restoring the wild-type phenotype, which implies that the sign of the behavioral response directly correlates with the state of the connectivity switch. In any case, further studies will provide a better understanding of the mechanism for signal transduction by the SRI-HtrI complex with CheA and CheW.

## 4. Bacterial Chemoreceptors

**Figure 8.**
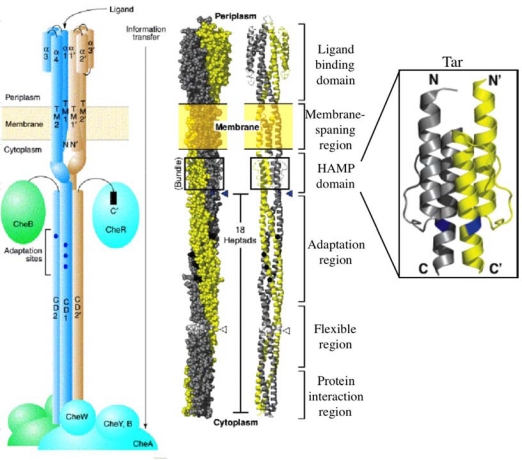
Structure of a dimeric bacterial chemoreceptor. (Left) Schematic illustration of the chemoreceptor. Each receptor monomer (∼60 kDa) consists of an N-terminal periplasmic ligand-binding domain, two transmembrane regions (TM1, TM2), a linker region and a C-terminal signaling/adaptation domain. Note that the HAMP domain structure is not integrated. (Center) Atomic structural model of the chemoreceptor generated by combining crystal structures of the fragments. The two symmetric subunits of the homodimer are shown as different colors. (Right) Deduced structure of the HAMP domain of the aspartate chemoreceptor Tar. Adopted from reference [140] and [145].

As described, MCPs act not only as receptors but also as transducers. Each MCP forms a homodimer regardless of its ligand occupancy state [20]. Each subunit (∼60 kDa) consists of, from the N-terminus to the C-terminus, the first transmembrane helix (TM1), the periplasmic ligand-binding domain, the second transmembrane helix (TM2), the HAMP domain and the kinase control module. The three dimensional structures of the homodimeric periplasmic domain of Tar both from *E. coli* and from *S. enterica* have been solved in the presence and absence of aspartate [129–132]. They showed that the periplasmic domain consists of a four helix bundle (Figure 8). The α1 and α4 helices of one subunit compose a membrane-spanning quasi-four-helix bundle with α1’ and α4’ of the partner subunit. Residues Arg64 (α1), Arg69 (α1’), Arg73 (α1’) and Thr154 (α4) of Tar are essential for the binding of aspartate [133–135]. The HAMP domain (the linker region), a 50 amino acid segment which is conserved among histidine kinases, adenylyl cyclases, MCPs and phosphatases [136–138], is thought to have a critical role in the signaling. Recently, the three-dimensional structure of a HAMP domain isolated from an unusual archeal membrane protein of unknown function was determined [139]. This archeal HAMP model has been confirmed in full-length membrane bound Tar by a disulfide cross-linking study with a series of cysteine-replaced mutant proteins [140]. The four helices of the HAMP domain are arranged in the same parallel, four-helix bundle architecture (Figure 8). The kinase control module has two long highly α-helical structures connected by a U-turn [141,142], which comprises three functional regions: the adaptation region possessing several methyl-accepting glutamate residues, a coupling region that transmits signals between other regions, and the protein interaction region, known as the highly conserved domain (HCD), which possesses contact sites for receptor oligomerization and for CheA-CheW binding (Figure 8). The HCD located around the tip of the kinase control module is highly conserved among MCPs from various eubacterial and archeal species [143,144]. Attractant binding to the MCP dimer induces a small but critical inward displacement (piston-like movement) of a membrane-spanning α-helix (TM2) of one subunit relative to a stable pair of TM1 and TM1’ [129,145–148]. This displacement is thought to be a trigger for a structural change in the kinase control module through the HAMP domain that inactivates CheA. HAMP is supposed to rectify the asymmetric piston inputs to a symmetric conformational change that modulates MCP output signals. It has been proposed that changes in helix-helix packing involves signaling through the four-helix bundle of the kinase control module and that the helix packing changes in the adaptation and protein interaction regions are tightly and antisymmetrically coupled (the yin-yang hypothesis) [149].

One of the remarkable features of bacterial chemotaxis is its high sensitivity. For instance, Tar has a very low threshold concentration (∼3 × 10^−8^ M) of l-aspartate for an attractant response [150–152]. Moreover, *E. coli* responds to very small changes (less than 1%) in the receptor occupancy with aspartate [153]. Thus, signals are not just transmitted but have to be amplified somewhere between input and output to produce a significant response. However, this amplification cannot be simply explained by the stoichiometric phospho-relay. At the motor level, cooperative interactions of phosphorylated CheY to FliM have been demonstrated but it is not sufficient to explain the unusually high Hill coefficient of the whole system [154,155]. The majority of the amplification is supposed to occur at the receptor level.

**Figure 9.**
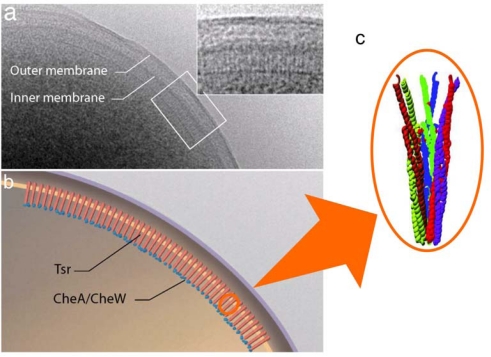
Visualization and identification of chemoreceptor arrays. (a) Low-dose cryo-projection image of the polar region in a wild-type *E. coli* cell, with the chemoreceptor array shown in greater detail in the *Inset*. (b) Schematic representation of the polar region of a wild-type E. coli cell illustrating the assembly and orientation of the chemotaxis receptor array, based on a and b. (c) The trimer of dimers of the cytoplasmic fragment of Tsr as a crystal unit. Adopted from reference [162] and [164].

MCPs localize to a cell pole, partially dependent on CheW and CheA, and form large clusters [156–163] (Figure 9). Each unit of the cluster is a “trimer of dimers” (hexamer), which contact at the cytoplasmic tip of the MCP [19,163–167] (Figure 9). The periplasmic architecture of the cluster is also a trimer of MCP dimers [168] (Figure 3). Altogether, MCP dimers are organized into a well-defined higher order array. This led to the proposal that attractant binding to a certain receptor dimer also affects the neighboring receptor dimer(s), a hypothesis which has been supported by several lines of evidence [165,169–177]. In fact, chemically synthesized multivalent ligands induce attractant responses of *E. coli* with lower thresholds than do the corresponding monovalent ligands [171,172]. These multivalent ligands are thought to promote receptor clustering to increase sensitivity. Thus, clustering of MCP is probably important for the chemotactic signaling system.

**Figure 10.**
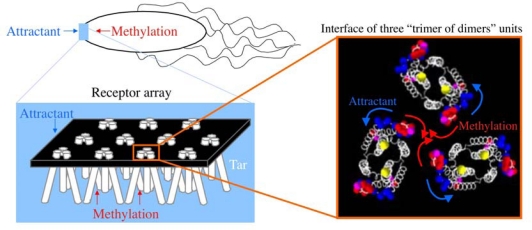
Model of gain control by covalent modification of receptors. At a cell pole, the chemoreceptors form clusters that are made of trimer of dimer units. The cytoplasmic interdimer interaction within trimers of dimers is thought to be important for the signaling. Our results newly demonstrate that receptor dimers interact at the periplasmic tips and these units are organized into a well-defined array. Two-state models of the chemoreceptor function assume two extreme states: one activating and the other inactivating CheA (kinase ON and OFF states, respectively). The efficiency of cross-linking at a given position is highest when the amidation state of the protein is QQQQ (red), EEEE (blue), or QEQE (magenta). Cross-linking at position 36 (yellow) is not detectably changed by amidation. The observed cross-linking is consistent with the notion that methylation (amidation) counteracts the attractant binding; attractant binding favors the OFF state, whereas methylation favors the ON state. The results also suggest that receptor methylation restricts the rearrangement (rotation) of the dimers by attractant binding (denoted by the blue arrows for the demethylated state and the red arrows for the methylated state), leading to a smaller gain for the same input signal. Adopted from reference [168].

In chemotaxis, adaptation is essential for the detection of temporal changes of stimuli and high sensitivity to stimuli over a wide dynamic range. Covalent modifications (*i.e.*, methylation and demethylation) of MCP are involved in the adaptation described above. The methylation of MCP slightly decreases the affinity of the receptor for its ligand [31,178–181] and slightly increases the CheA activity [31,181]. However, such changes are too small to account for the adaptation. Rather, the methylation seems to control signal gain to cause significant adaptation [173,181]. In fact, an *in vivo* disulfide cross-linking study suggested that methylation (amidation) of Tar and the attractant binding to Tar dimers might alter the arrangement of those dimers in roughly opposite directions (possibly inducing rotational movement) without much affecting the conformation of the periplasmic domain in a pre-formed cluster [168] (Figure 10). Cross-linking analyses with chemical cross-linkers with different arm lengths brought about a consistent and more detailed estimation of the arrangement of Tar dimers in the cluster (K. Jintori, H.I. and I.K, unpublished results). Furthermore, increasing levels of methylation (amidation) attenuates the effects of attractant binding. These results are consistent with the “two-state models” of receptor signaling and adaptation [182–186]. In essence, the receptor array is in an equilibrium between the kinase ON and OFF states, which are favored by methylation (amidation) and attractant binding, respectively, and the attenuation of attractant effect by receptor methylation implies the down-regulation of signal gain, suggesting that the gain control by covalent modification of the chemoreceptor involves modulation of the arrangement or packing of the receptor array. Thus, these results permit us to speculate that the rotational motion of photo-transducer and chemo-transducer proteins are essential movements of the proteins required for the signal transduction.
